# Concepts for structured illumination microscopy with extended axial resolution through mirrored illumination

**DOI:** 10.1364/BOE.382398

**Published:** 2020-03-20

**Authors:** James D. Manton, Florian Ströhl, Reto Fiolka, Clemens F. Kaminski, Eric J. Rees

**Affiliations:** 1 Department of Chemical Engineering & Biotechnology, University of Cambridge, CB3 0AS, UK; 2 MRC Laboratory of Molecular Biology, Francis Crick Avenue, Cambridge, CB2 0QH, UK; 3 Present address: Department of Physics and Technology, UiT The Arctic University of Norway, NO-9037 Tromsø, Norway; 4 Department of Cell Biology, UT Southwestern Medical Center, 6000 Harry Hines Blvd., Dallas, Texas 75390, USA; 5 Lyda Hill Department of Bioinformatics, UT Southwestern Medical Center, 6000 Harry Hines Blvd., Dallas, Texas 75390, USA

## Abstract

Wide-field fluorescence microscopy, while much faster than confocal microscopy, suffers from a lack of optical sectioning and poor axial resolution. 3D structured illumination microscopy (SIM) has been demonstrated to provide optical sectioning and to double the resolution limit both laterally and axially, but even with this the axial resolution is still worse than the lateral resolution of unmodified wide-field microscopy. Interferometric schemes using two high numerical aperture objectives, such as 4Pi confocal and I^5^M microscopy, have improved the axial resolution beyond that of the lateral, but at the cost of a significantly more complex optical setup. Here, we theoretically and numerically investigate a simpler dual-objective scheme which we propose can be easily added to an existing 3D-SIM microscope, providing lateral and axial resolutions in excess of 125 nm with conventional fluorophores and without the need for interferometric detection.

## 1. Introduction

Over the past three decades, there has been a considerable effort to improve the spatial resolution of fluorescence microscopy, with the majority of advances being focussed on improving the lateral resolution [1–16]. However, in many cases, improving the axial resolution would be just as, if not more, beneficial, particularly as the axial resolution of a conventional microscope is much worse than its lateral resolution. While both lateral and axial resolution increase with numerical aperture (NA), even an ideal single objective system that could collect over a full hemisphere on one side of the sample is bound to have an axial resolution at best half that of the lateral resolution. Such anisotropic resolution is highly detrimental for the accurate quantification of object sizes, shapes, volumes and curvatures.

3D structured illumination microscopy (3D-SIM) has been shown to double both lateral and axial resolution in wide-field microscopy through patterned illumination generated by the interference of three beams [17]. well as developments in improving temporal resolution [18,19], it has successfully been combined with dual-objective interferometric illumination and detection in I^5^S microscopy to achieve an unprecedented wide-field 3D isotropic resolution of 90 nm [20,21]. Despite this impressive feat, use of this technology has been extremely limited due to the experimental difficulties in constructing and operating such a system. Even the use of the related dual-objective confocal technique of 4Pi microscopy has been limited by its complexity, with most existing systems relying on the simplest of the three 4Pi methods, Type A, in which only illumination light from both objectives is made to interfere [22].

The main difficulty arising in implementing Type C 4Pi or I^5^S microscopy, in which both illumination and emission light are made to interfere, is ensuring that the path lengths from sample to beamsplitter in the interferometric arms are equal to within the coherence length of the fluorescence light (i.e. a few microns). In contrast, the relatively long coherence lengths of lasers commonly used in fluorescence microscopy ensures that interfering the excitation light, as in Type A 4Pi microscopy, is comparatively straightforward.

In this article, we briefly review the theory of 3D-SIM and use this to propose a simple dual-objective scheme that does not require interferometric detection to further improve the achievable axial resolution. In addition, by relaxing the technical constraints on the secondary objective, our scheme is compatible with low NA, high working distance objectives, further simplifying an experimental realisation.

We first review the theory of operation of 3D-SIM and use this to introduce our method. We present an analysis of the potential performance of our approach via geometric considerations and support this with numerical simulations of the image formation process, providing examples of expected images given a known ground truth object. We detail the practical considerations for an experimental realisation of our method and propose that existing 3D-SIM microscopes can be easily modified to further double axial resolution while maintaining the same reconstruction approach.

### 1.1. 3D-SIM theory

Considering a fluorescence microscope as a linear, shift-invariant imaging system, the image data collected, D, can be considered as the convolution of the fluorescence emission, F, with a point spread function (PSF), H, such that D=F∗H=(S×I)∗H, where S is the sample fluorophore distribution and I is the illumination intensity distribution. Alternatively, in the Fourier domain, $\tilde{D}=\tilde{F}\times \tilde{H}=(\tilde{S}*\tilde{I})\times \tilde{H},$ where overset tildes denote the Fourier transforms of the respective real-space functions and $\tilde{H}$ is the optical transfer function (OTF) [5–7].

**Fig. 1.**
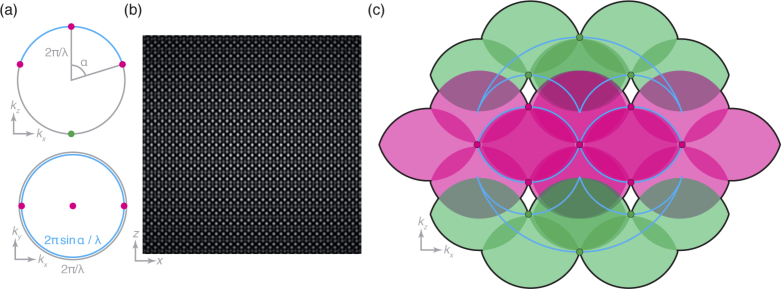
Creating an illumination profile using four mutually coherent beams. (a) Fourier domain illumination electric field distribution formed by four coherent beams, with those present in 3D-SIM drawn in magenta and the additional beam considered in this work drawn in green. Here the angular separation is the maximum possible, namely the half-angle, α, of the lens. (b) Real domain illumination intensity distribution resulting from the electric field distribution of (a). (c) Fourier domain illumination intensity distribution drawn as magenta and green spots (corresponding to those present in 3D-SIM and the new components considered in this work) with the overall support of the OTF drawn as a black line and each wide-field OTF contribution shown in the colour of the illumination component that produced it. These illumination components can be seen to lie on the edge of the support of the 4Pi OTF (cyan lines) for this case where the angular separation is α. Note the holes within the support of the OTF near the off-axis green spots.

The illumination intensity, I, is related to the electric field, E, via $I=EE^{*}$, where $E^{*}$ is the complex conjugate of E. Hence, the Fourier domain distribution, $\tilde{I}$, is given by the autocorrelation of $\tilde{E}$. In a conventional 3D-SIM system, the illumination is generated by three interfering laser beams produced by the 0 and ±1 diffraction orders of a grating. This means that the Fourier domain electric field amplitude distribution, $\tilde{E}$, consists of three points on a shell of radius 2π/λ, spaced angularly by no more than α, where λ is the wavelength of the illumination light (given by $\lambda_{0}/n$, where $\lambda_{0}$ is the vacuum wavelength and n is the refractive index of the medium) and α is the half-angle of the objective lens (shown in magenta in Fig. 1(a)). The Fourier domain distribution, $\tilde{I}$, hence consists of the seven magenta points shown in Fig. 1(c) [17].

The detection system of the microscope cannot capture information outside the support of the OTF, but this illumination structure down-mixes higher spatial frequency information into the passband of the detection objective. Under the assumption that the illumination pattern remains fixed with respect to the objective as z-stacks are collected, it has previously been shown by Gustafsson et al. that the components are already unmixed axially by the process of moving the sample through focus [17]. All that remains is to unmix laterally, by applying lateral phase shifts to the illumination pattern, producing as many bands of information as there are columns of components in the illumination Fourier domain distribution. In this case, while there are seven components in total (magenta points in Fig. 1(c)), only five phase steps are required. By repeating this process for multiple orientations of the illumination pattern, the lateral resolution enhancement is made approximately isotropic. Typically three orientations are used, requiring 15 images per focal plane. We can consider the effective overall OTF as the combination of all the axially extended bands produced by this process, shown with a magenta fill in Fig. 1(c).

## 2. Results

### 2.1. Further improving axial resolution in SIM by including a fourth illumination beam

Consider a modified 3D-SIM system in which a further central beam is incident upon the sample from the opposite side. Here, the Fourier domain electric field amplitude distribution includes an additional point on the shell of radius 2π/λ opposite the original central beam (green point in Fig. 1(a)). This causes the Fourier domain illumination intensity distribution to gain a further six points, drawn in green in Fig. 1(c).

As before, we can describe the effective overall OTF as the combination of all the axially extended bands produced by this process, the bandlimit of which shown as the black line in Fig. 1(c). Here, six copies of the wide-field OTF (shown in green) have been added above and below the original 3D-SIM OTF, providing enhanced optical sectioning and axial resolution. Because the additional beam is incident on-axis, no extra lateral frequency components are created. This means that the lateral unmixing process still requires only five phase shifts, but as the axial support of the OTF is enlarged z-stacks must be acquired with finer steps in order to satisfy the Nyquist sampling criterion.

### 2.2. Geometric considerations for filling the holes in the OTF

Taking the 13-point illumination structure and convolving with the conventional wide-field OTF produces a system OTF with larger axial support but which, crucially, contains holes (white fill in Fig. 1(c)). While considerably smaller than those present in standing-wave microscopy, in order for all sample frequencies within the maximum extent of the OTF to be accurately recorded these holes must be filled [23]. This cannot be achieved by merely changing the angular spread of the outer points of the illumination and so alternative approaches must be sought. Here, we consider three approaches to achieve this. In the following discussion, examples are shown for an oil-immersion 1.45 NA lens operating in a nominal refractive index of 1.518. Unless otherwise stated, illumination is at 488 nm with fluorescence emission detected at 510 nm.

#### 2.2.1. Illuminating with multiple wavelengths

**Fig. 2.**
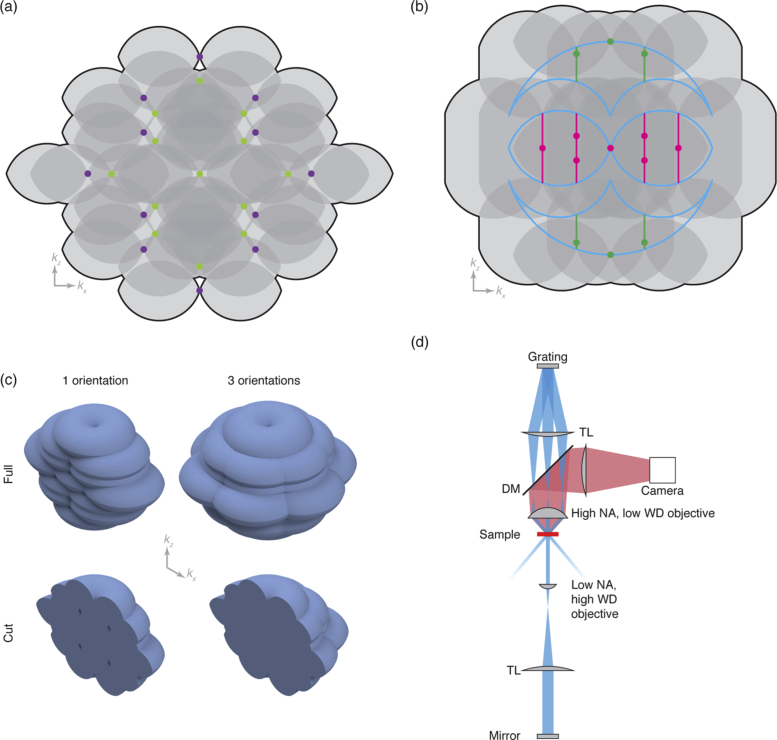
Strategies for filling the holes in the OTF. (a) Overall effective OTF (support shown as black line) for illumination at 445 nm (purple spots) and 561 nm (yellow-green spots) with fluorescence detection at 610 nm (gray OTFs). Here the holes are filled due to the difference in k-vector length, but two new ones are created along $k_{z}$. These can easily be removed by apodisation at the cost of slightly reduced resolution. (b) The effect of multimode illumination with a pupil fraction of 1/3. Considering the centre of each illumination image created by the grating leads to the Fourier domain illumination distribution shown as the magenta and green spots. Summing over all such point tetrads causes an axial broadening of this distribution, shown as the magenta and green lines. While no contributions from outside the 4Pi OTF exist, the shifting of contributions within this ensures a good overlap and hence no holes in the overall OTF support. (c) The effect of considering multiple orientations on the $k_{x}$–$k_{z}$ coverage. With one orientation, a cut through the full 3D OTF support shows the holes suggested by the 2D geometrical consideration. However, with this 3D view it becomes clear that these holes are filled by contributions from the other orientations when three orientations are used to produce the full 3D OTF. (d) Schematic of the proposed experimental setup in which a conventional 3D-SIM microscope is augmented with a low numerical aperture, high working distance objective on the other side of the sample, paired with a tube lens and mirror to reflect just the central beam.

Consider the effect of illuminating with two different wavelengths. While the overall structure of the illumination will be similar in each case, the wavelength difference will cause the distance from the origin of each Fourier domain illumination component to differ (compare purple and green points in Fig. 2(a)). By selecting an appropriate combination of wavelengths, such as 445 nm and 561 nm illumination with 610 nm detection, the holes for each wavelength can be filled with information obtained using the other illumination wavelength. With this, nine phase steps rather than five are required (as there are nine distinct columns of components), slowing acquisition speed. In addition, the structure of interest now needs to be labelled with fluorophores with a broad excitation spectrum, such as quantum dots, complicating multicolour imaging of different structures. Alternatively, two different fluorophores with well-separated excitation and emission spectra can be used, with two sets of five-phase data acquired and fused, at the expense of a further reduction in acquisition speed. A further potential pitfall occurs if the modulation contrast of the two colours is different — a lower modulation contrast for one colour would lead to weaker bands, producing a less uniform overall OTF.

#### 2.2.2. Illuminating with multiple modes

Instead of illuminating with multiple wavelengths, consider illuminating not with single modes in the pupil, but with multiple independent incoherent modes over a significant area of the pupil. Each order generated by the grating produces an image of the source in the pupil. For appropriately spatially incoherent illumination, each point within each source image will be incoherent with all other points within the same image, but coherent with the corresponding points in the other source images [17].

As the objective lens obeys the Abbe sine condition, points in the pupil are projected orthographically onto the shell of radius 2π/λ when considering the Fourier domain electric field amplitude distribution. Hence, for each set of source point images, the lateral components of the Fourier domain illumination intensity distribution will not change while the axial ones will. The end effect, after summing over all points within the source images, is an illumination intensity distribution in which each of the original points (except for ones along $k_{z}$) have been broadened axially but not laterally (green and magenta lines in Fig. 2(b)).

The outermost components are broadened to the full axial extent of the wide-field OTF at that lateral frequency, while the inner components are broadened half as much (see Visualization 1). For a pupil fraction of 1/3 (the maximum possible before source images overlap), corresponding to a maximum lateral illumination frequency of 2/3 that of the maximum supported by the lens, the effect is such that all components are broadened to the full extent of the local component of the dual-objective OTF (compare green and magenta lines with cyan dual-objective OTF extent in Fig. 2(b)).

While the exact absolute extent of the axial broadening and overlap is dependent on the exact numerical aperture, refractive index of immersion, n, and pupil fraction used, a pupil fraction of 1/3 is more than sufficient to fill all the holes for a 1.45 numerical aperture oil immersion objective (n=1.518) as demonstrated in Fig. 2(b). While this approach does not require any changes to the sample labelling and fills the holes with single-orientation reconstruction procedures, the maximum possible lateral resolution enhancement is decreased as the illumination pattern is formed by beams spaced by a lower numerical aperture.

Furthermore, while high lateral modulation contrast is maintained in the illumination pattern, the relative strengths of the separated Fourier domain bands is weakened with respect to the central band in 3D. This is because each coherent point tetrad in the pupils provides a Fourier domain illumination intensity component at the origin irrespective of the tetrad location in the pupils. Hence, using a larger pupil fraction strengthens the central band at the expense of the others as these are further elongated axially. A 2D demonstration of this effect is shown in Visualization 1.

#### 2.2.3. Recording data with multiple pattern orientations

So far we have only considered a two-dimensional system operating in the xz-plane (where z is the optical axis). In a true three-dimensional system the wide-field OTF is not the bi-lobed object depicted in cyan in Fig. 1(c), but the toroidal solid of revolution given by that cross-section [24]. Hence, by recording data with three pattern orientations spaced by 120°, the holes in one orientation are filled by contributions from the others (as shown in Fig. 2(c)). Therefore, illuminating with multiple wavelength or with multiple modes is not strictly necessary if the numerical aperture of the detection objective is sufficiently large. However, this precludes acquisitions using just one orientation, which may be desired for reasons of speed when enhancing the axial resolution is more important than an isotropic lateral resolution enhancement.

### 2.3. Numerical simulations of imaging performance

**Fig. 3.**
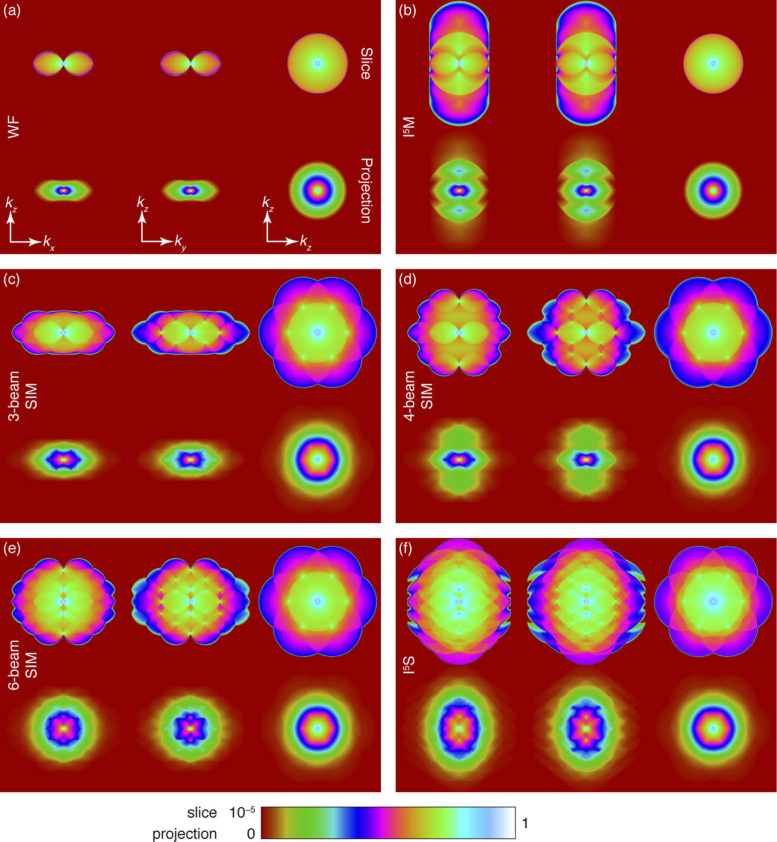
Simulated modulation transfer functions (MTF) for wide-field microscopy techniques, assuming an aperture half-angle of 72.7°. Top rows show the central slice of the MTF, while bottom rows show the projection of the MTF (as would be seen by Fourier transforming a slice of a sub-diffraction bead). All panels follow the same co-ordinate systems. Central slices are shown on a logarithmic scale to enhance dim details. Visualization 3 shows a flythrough of these same MTFs. (a) Wide-field microscopy with uniform illumination. (b) I^5^M (incoherent illumination + interferometric detection). (c) 3D-SIM. (d) Our proposed dual-objective four-beam SIM (without interferometric detection). (e) Dual-objective six-beam SIM without interferometric detection. (f) I^5^S (dual-objective six-beam SIM + interferometric detection).

In order to validate our conclusions based on a geometrical understanding of the optical transfer functions relevant to our proposed approach, we produced numerical simulations of imaging performance based on a scalar, non-paraxial model of diffraction. Figure 3 shows simulated modulation transfer functions for conventional wide-field microscopy, I^5^M [25], 3D-SIM, our proposed approach, six-beam structured illumination microscopy (i.e. I^5^S without interferometric detection), and I^5^S microscopy.

Detection OTFs were produced by autocorrelating caps of the Ewald sphere, assuming an aperture half-angle of 72.7° (corresponding to a 1.27 NA water immersion objective or a 1.45 oil immersion objective for index-matched samples). The illumination intensity Fourier distribution was produced by autocorrelating appropriate sections of the Ewald sphere, with the result being Fourier transformed to produce a real space illumination function. For structured illumination techniques, the ±1 orders were set to lie at 95 % of the pupil radius, with three orientations of the pattern used to generate the overall illumination function. The overall OTF was produced by multiplying the detection point spread function (produced by Fourier transforming the calculated OTF) with the illumination function and Fourier transforming the result.

In the displayed results, OTFs labelled ‘slice’ correspond to a slice of the OTF in the Fourier domain, not a slice of the image data in the real domain. Similarly, OTFs labelled ‘projection’ correspond to a sum of the OTF along the unshown axis (i.e. orthogonal to the page) and so, by the projection-slice theorem, correspond to a slice of the image data in the real domain (i.e. these are the equivalent to the result of Fourier transforming the image data corresponding to a slice of a sub-diffraction point source). Code to reproduce these results is presented in Supplementary Code 1 [26].

The OTFs shown in Fig. 3(d) support our conclusion in Section 2.2.3 that multiple orientations alone are sufficient to fill the holes in the OTF, without the need for illumination at two wavelengths (Section 2.2.1) or with multiple modes (Section 2.2.2). Indeed, here the improvements in optical sectioning and axial resolution are clearly shown by the high value of the transfer function along $k_{z}$ away from the origin — only at the very edge of the support does the value fall below that shown in the corresponding lateral transfer function at the usual diffraction limit in the 3D-SIM case (see Fig. 3(c)). Visualization 2 shows the effect of increasing the primary objective NA on the OTF and clearly shows that the holes are well-filled by a 1.2 NA water immersion lens (or, equivalently, a 1.37 NA oil immersion lens). Indeed, the value of the transfer function in the ‘hole’ region is shown to be larger than the value near high lateral frequencies — these latter values are already sufficiently high for resolution enhancement in 3D-SIM and so we conclude that the holes are well-filled by the different orientations. Experimentally, OTFs are always found to be weaker than theory suggests, supporting the idea that the holes should be well-filled in our simulations in order for them to be filled in practice. In addition, we see that the holes are not filled at an NA of 1.1, the highest commercially available NA for a water-dipping objective.

**Fig. 4.**
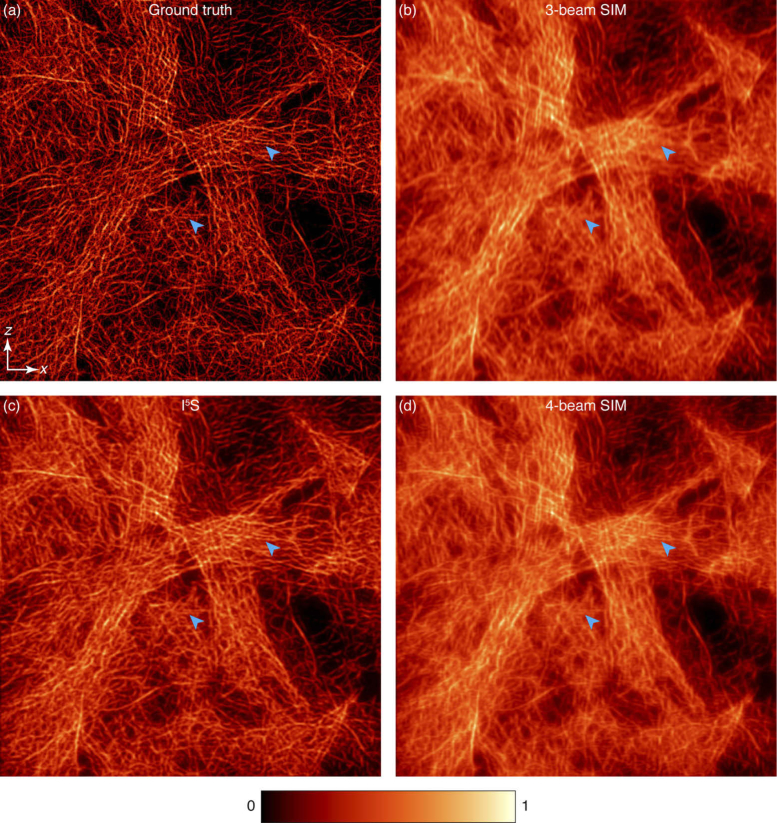
Simulated imaging performance on a fibrous ground truth test image, shown as an xz slice. All images are normalised to have the same minimum and maximum intensity. Cyan arrowheads point to closely-spaced fibre pairs that cannot be resolved using conventional 3D-SIM, but which are resolvable using our four-beam approach or I^5^S. (a) Ground truth. (b) 3D-SIM. (c) I^5^S (dual-objective six-beam SIM + interferometric detection). (d) Our proposed dual-objective four-beam SIM (without interferometric detection).

To more readily appreciate the expected improvement in optical sectioning and axial resolution given by our technique over 3D-SIM, we used these OTFs to produce simulated images given a constructed ground truth (see Fig. 4). Here, an xz slice of an artificial ground truth structure is shown in Fig. 4(a). Corresponding image data slices for 3D-SIM, I^5^S, and our proposed four-beam SIM method are shown in Fig. 4(b–d). These were produced by filtering a ground truth sample structure with the respective OTF in the Fourier domain and Fourier transforming the result to produce a real space image. Here, we have purposefully neglected to include the effects of noise to more clearly show the effects of the respective transfer functions.

First, we note that the I^5^S image shows the best contrast, as expected from its more uniform transfer function. Second, we note that both 3D-SIM and our proposed method have similar contrast, but that our method clearly separates closely-spaced fibrilar structures that are resolved as one thicker object in the 3D-SIM case (cyan arrowheads in Fig. 4). Finally, while the resolution of I^5^S is theoretically superior to our proposed method due to its larger axial support, it is hard to appreciate this in the raw image data, suggesting that our method may provide almost all of the practical resolution improvements of I^5^S despite its relative simplicity.

### 2.4. Considerations for an experimental realisation

While the required fourth beam could be provided via an interferometer-like arrangement, where a beamsplitter and steering mirrors direct some of the illumination light to the secondary objective, the required fourth beam can also be provided via a relatively simple modification to an existing 3D-SIM system. By placing a low numerical aperture, high working distance objective on the opposite side of the sample, the central beam can be captured while the outer beams are left to escape. A tube lens and mirror placed in the image plane reflects this central beam, sending a fourth beam into the sample as required (see Fig. 2(d)). While there will be some loss of intensity of this reflected beam, leading to weaker contributions in the Fourier domain illumination intensity distribution (green components in Fig. 2(c)), we would still expect >75% of the maximum possible contribution given typical transmittance and reflectance values. Furthermore, this scheme provides the pupil mapping necessary for the multimode approach by default, rather than relying on extra beam expansion and careful objective choice required for the interferometer-like arrangement.

As we require this reflected beam to interfere with the three incident from the high NA objective side of the sample, the coherence length of the laser must be greater than the ~800 mm extra path length experienced by the reflected beam. Hence, for a 488 nm laser line, the frequency bandwidth must be less than ~100 MHz, assuming a Lorentzian lineshape. While laser sources conventionally used for SIM may not have a sufficiently small bandwidth, appropriate single frequency lasers (such as the Coherent Sapphire SF 488, with a 1.5 MHz linewidth) are readily available from commercial sources.

In order for the central beam to be fully captured by the low NA objective, the NA of this lens must be greater than the product of the NA of the high NA objective with the illumination pupil fraction. Objective lenses with NAs of 0.5 and working distances greater than 1 mm are commercially available and would be suitable for capturing the central beam from a 1.45 NA primary objective, even if the pupil fraction is the maximum possible (1/3).

If the multimode approach is to be used, illumination can be provided by laser light coupled into a shaken multimode fibre as this well-approximates a spatially incoherent source over sufficiently long times (typically of the order of less than a millisecond) [19]. As the diameter of the objective pupil is given by 2f×NA, where f is the back focal length and NA is the numerical aperture, a 1/3 pupil fraction would correspond to a ~20-fold magnification for a 100 µm core fibre and a 100× objective designed for a 200 mm tube length. Such fibres are commonly available with a numerical aperture of 0.22, ensuring minimal loss of light through the relay optics from fibre tip to pupil image while still satisfying the conditions for proper demagnification of the grating onto the sample.

Practically, only a small pupil fraction would be necessary, with the multimode illumination being used mainly to reduce laser speckle and other coherence artefacts while the multiple pattern orientations fill the holes in the OTF. With this in mind, we can consider using a water-dipping lens as the secondary objective. This would make our method compatible with imaging samples loaded into coverglass-bottomed wells, with easy access to the sample for electrophysiology, treatment delivery and media exchange maintained. This is in contrast to the existing 4Pi confocal, I^5^M and I^5^S methods which require the sample to be mounted within a coverglass sandwich as they require high-NA immersion lenses.

## 3. Discussion

We have investigated the possibility of further axial resolution enhancement in 3D-SIM by reflecting the central beam and have provided methods for countering the existance of holes in the OTF support. To our surprise, we found that a fully three-dimensional consideration of the image formation process in structured illumination microscopy showed that these holes are well-filled when a full set of image data for all (three) pattern rotations are acquired and combined. Numerical simulations demonstrated a clear improvement in axial resolution over 3D-SIM, with a practical effective resolution close to that of I^5^S.

For an objective with an aperture half-angle of 72.7° (corresponding to a 1.27 NA water immersion objective or a 1.45 oil immersion objective for index-matched samples) a fully isotropic 3D resolution (as defined by OTF support) at ~1.5× the diffraction-limited lateral resolution can be achieved by apodising a fully reconstructed four-beam SIM dataset using three pattern orientations. Ignoring a further resolution enhancement provided by the Stokes’ shift, this would convert a live-imaging system at 510 nm from a 200nm×200nm×545nm resolution to a 135nm×135nm×135nm resolution, i.e. an almost nine-fold increase in volumetric information density.

While further resolution enhancements would be possible by using another high NA lens as the secondary objective to provide a further two illumination beams, and/or by using interferometric detection as in I^5^M and I^5^S microscopy [20,25], the comparatively simple experimental setup proposed here achieves much of the performance of such a system while avoiding the significant complexity of interferometric detection. By relaxing the requirements of the secondary objective, our approach is compatible with imaging samples mounted in a conventional manner in multi-well plates, rather than sandwiched between two coverslips — a long-working-distance dipping lens is a sufficient secondary objective.

In addition to having the same dependence on polarisation purity as in 3D-SIM, we emphasise that our approach is compatible with existing methods for reconstructing three-dimensional structured illumination microscopy data, with a minor modification to the shape of the OTF bands used. These are readily calculated or measured using exactly the same approaches as used for conventional 3D-SIM and so should be straightforward to integrate into existing sofware, such as fairSIM [27].
